# Dosimetric and radiobiological comparison in head-and-neck radiotherapy using JO-IMRT and 3D-CRT

**DOI:** 10.1016/j.sjbs.2022.103336

**Published:** 2022-06-02

**Authors:** Duong Thanh Tai, Luong Thi Oanh, Pham Hoai Phuong, Abdelmoneim Sulieman, Fouad A. Abolaban, Hiba Omer, James C.L. Chow

**Affiliations:** a Department of Medical Physics, Faculty of Medicine, Nguyen Tat Thanh University, 298-300A Nguyen Tat Thanh Street, Ward 13, District 4, Ho Chi Minh City, Viet Nam; b Robarts Research Institute, London, Ontario, N6A 5K8, Canada; c Department of Radiation Technology, Ho Chi Minh City Oncology Hospital, Ho Chi Minh City 702000, Viet Nam; d VKTECH Research Center, Nguyen Tat Thanh University, 298-300A Nguyen Tat Thanh Street, Ward 13, District 4, Ho Chi Minh City, Viet Nam; e Prince Sattam Bin Abdulaziz University, College of Applied Medical Sciences, Radiology and Medical Imaging Department, Alkharj, Saudi Arabia; f Department of Nuclear Engineering , Faculty of Engineering, King Abdulaziz University P. O. Box 80204, Jeddah 21589, Saudi Arabia; g K. A. CARE Energy Research and Innovation Center, King Abdulaziz University, Jeddah 21589, Saudi Arabia; h Department of Basic Sciences, Deanship of Preparatory Year and Supporting Studies, Imam Abdulrahman Bin Faisal University, P.O.Box 1982, Dammam 34212, Saudi Arabia; i Department of Radiation Oncology, University of Toronto, Toronto, ON, M5S 1A1, Canada; j Radiation Medicine Program, Princess Margaret Cancer Centre, University Health Network, Toronto, ON, M5G 1X6, Canada

**Keywords:** 3D-CRT technique, JO-IMRT technique, Dosimetric and radiobiological evaluation, Equivalent Uniform Dose (EUD), Plan evaluation, JO-IMRT, Jaws-only Intensity-modulated radiotherapy, 3D-CRT, 3D conformal radiation therapy, CI, Conformity index, HI, Homogeneity index, TCP, Tumor control probability, NTCP, Normal tissue complication probability, PTV, Planning target volume, EUD, Equivalent uniform dose

## Abstract

**Introduction:**

Dosimetric and radiobiological evaluations for the Jaws-only Intensity-modulated radiotherapy (JO-IMRT) technique for head and neck jaws-only intensity-modulated radiation therapy (JO-IMRT) and 3D conformal radiation therapy (3D-CRT). To compare the head-and-neck therapeutic approaches utilizing JO-IMRT and 3D-CRT techniques, different radiation dose indices were calculated, including: conformity index (CI), homogeneity index (HI), and radiobiological variables like Niemierko's equivalent uniform dose based tumor control probability (TCP) of planning target volume (PTV), normal tissue complication probability (NTCP) of organs at risk (OAR) (brainstem, spinal cord, and parotid grand).

**Materials and methods:**

Twenty-five nasopharynx patients were studied using the Prowess Panther Treatment Planning System (Prowess Inc). The results were compared with the dose distribution obtained using 3D-CRT.

**Results:**

Regarding tumor coverage and CI, JO-IMRT showed better results than 3D-CRT. The average doses received by the PTVs were quite similar: 72.1 ± 0.8 Gy by 3D-CRT and 72.5 ± 0.6 Gy by JO-IMRT plans (p > 0.05). The mean doses received by the parotid gland were 56.7 ± 0.7 Gy by 3D-CRT and 26.8 ± 0.3 Gy by JO-IMRT (p > 0.05). The HI and CI were 0.13 ± 0.01 and 0.14 ± 0.05 and (p > 0.05) by 3D-CRT and 0.83 ± 0.05 and 0.73 ± 0.10 by JO-IMRT (p < 0.05). The average TCP of PTV was 0.82 ± 0.08 by 3D-CRT and 0.92 ± 0.02 by JO-IMRT. Moreover, the NTCP of the parotid glands, brain stem, and spinal cord were lower using the JO-IMRT than 3D-CRT plans. In comparison to the 3D-CRT approach, the JO-IMRT technique was able to boost dose coverage to the PTV, improve the target's CI and HI, and spare the parotid glands. This suggests the power of the JO-IMRT over 3D-CRT in head-and-neck radiotherapy.

## 1 Introduction

Malignant tumors are the leading cause of death and a serious public health concern around the world (Bray et al., 2021). According to the latest stats, cancer affects 19.3 million people worldwide each year, with 50% of patients dying from cancer and around 70% of cancer fatalities occurring in middle- and low-income nations (Sung et al., 2021). Cancer cases are predicted to rise as a result of changes in lifestyle, demographics, and economic growth, an increase of lifespan as well as increased cancer risk factors. Almost 19.3 million cancer cases were reported in 2020, 10 million of whom died. (Sung et al., 2021) distribution of cancer cases worldwide depends mainly on the type of cancer, age, gender, etc.

Head and neck cancers develop in or around the mouth and oral cavity and salivary glands, nose, nasal cavity, and sinuses as well as the throat and larynx as well as the surrounding skin. Squamous cell carcinoma is the most common type. They do not include all the tumors that exist in the head and neck area, for instance brain, eyes, and thyroid.

Treatment for cancer is a difficult task. To treat cancer, a single or a mix of therapy modalities may be required including surgery, radiosurgery, radiotherapy chemotherapy, and hormonal therapy. Each of these modalities has its curative as well as harmful effects on the patients and thus implementation with high accuracy and precision is necessary.

Radiation of the tumor and tumor bed applied pre-during-and post-surgery used high doses of ionizing radiation to the PTV. The purpose of this treatment is to give the tumor the highest and most lethal dose of radiation while preserving the healthy tissues (Khairi et al., 2021, Zugazagoitia et al., 2016). Intensity modulated radiotherapy (IMRT) proved to fulfill the aim of treatment very effectively and is hence widely applied to treat cancer today. There are two kinds of IMRT techniques: (1) Multi-leaf collimators IMRT (MLC-IMRT) and (2) Jaws-only IMRT (JO-IMRT). The JO-IMRT technique employs the integrated jaws of the medical linear accelerator (LINAC). This technique was implemented on the Prowess Panther treatment planning system (TPS). On the other hand, the 3D-CRT technique is still being widely used. In fact, this technique causes many unwanted complications to patients.

Radiation treatment planning plays an essential role in treating cancer. There are many situations in the radiotherapy (RT) planning procedure that plans based on different dose delivery techniques should be compared to select the best treatment plan (Puzhakkal et al., 2016, Lee et al., 2015, Ebert, 2001).

Numerous ways of assessment of a particular therapy treatment plan exist; including but not limited to: assessment via calculation of conventional-dose indices, such as the conformity index (CI), homogeneity index (HI), and radiobiological parameters like Niemierko's EUD-based tumor control probability (TCP), and normal tissue complication probability (NTCP) (Chow et al., 2019, Khan et al., 2016, Kim et al., 2015, Paddick, 2000).

The comparison of multi-types of radiotherapy plans using the radiobiological evaluation has been published in research (Puzhakkal et al., 2016; Chow et al., 2020, Rehman et al., 2018, Kim et al., 2018, Paudel et al., 2017). In 2016, Puzhakkal et al. employed radiobiological techniques to confirm the treatment of 30 patients with head and neck and brain tumors as well as prostate cancers. Limited statistically significant differences existed in either biological or physical dosage indices of the healthy nearby organs. Paudel et al. examined IMRT and VMAT plans for quality assurance using the assessment method (Paudel et al., 2017). They suggested that the radiobiological analysis should be considered for the assessment of complex radiotherapy plans. Khan et al. on the other hand, explored characterizations of IMRT and VMAT for prostate cancer using both radiobiological and dosimetric parameters (Khan et al., 2016). Chow et al. compared different algorithms using the dosimetric and radiobiological parameters of VMAT plans for the prostate (Chow et al., 2020). Chow & Jiang evaluated the different doses in calculation grid size by using the dose-volume and radiobiological in VMAT planning for prostate (Chow & Jiang, 2018). Hence, a radiobiological parameter is helpful for many different purposes.

In Vietnam, the 3D-CRT technology is widely used in clinical practice (Tai et al., 2017a). The JO-IMRT technology was recently used for the first time in Vietnam's Dong Nai General Hospital. (Tai et al., 2017b). While the scientific paper that compares IMRT and VMAT technique in prostate cancer was previously published, to our knowledge, there are very few studies that report a dosimetric comparison of JO-IMRT and 3D-CRT techniques for the head & neck tumors implemented in the Prowess Panther TPS (Tai et al., 2017b, Tai et al., 2017, Tai et al., 2019, Tai et al., 2018). Because the JO-IMRT technology is still pretty recent and not extensively used, there are just a few studies on it. As a result, this study evaluated the dosimetric and radiobiological properties of Prowess Panther TPS's 3D-CRT and JO-IMRT techniques.

## 2 Materials and methods

### 2.1 Treatment plans

The 3D-CRT and JO-IMRT treatment plans for 25 nasopharynx patients were established using the Prowess panther TPS (Panther, Prowess Inc., Chico, CA). Two parallel opposing tangential 6 MV photon beams generated by Siemens Primus LINAC (Siemens Medical Solutions, Concord, CA) were employed in the 3D-CRT plans to determine the dose distribution using a rapid photon effective technique, whereas seven coplanar intensity-modulated beams were used in the JO-IMRT plans. For the following fixed gantry angles, a step-and-shoot module was used: 0, 50, 100, 150, 200, 250, and 300 degrees. The dose distributions produced by JO-IMRT were calculated using the collapsing cone convolution technique. The dosage was 66 Gy in 30 fractions, covering >95% of the planning target volume (PTV). The RTOG-0022 protocol was utilized for the crucial structural approval criteria (Eisbruch et al., 2005).

### 2.2 Plan evaluation

#### 2.2.1 Dosimetric analysis

To evaluate the treatment plan, dosimetric factors for instance: dose and dose-volume parameters are commonly used (Banaei et al., 2019). The evaluation process comprises two steps: (1) considering the dose distributions on the computed tomography image from the TPS; and (2) examining the dose-volume histograms (DVHs) for the mean and maximum dose for the PTV and organs-at-risk. Although DVH displays dose-volume coverage data, it does not provide other geometric data such as the position of the hot spot or dose homogeneity (Park et al., 2014). For this reason, the homogeneity index (HI) was utilized for plan appraisal. The International Commission on Radiation Units and Measurements Report 83 defines HI as a measure of dose uniformity over the PTV (ICRU, 2010):

(1) $$\mathrm{HI}=\frac{D_{2\%}-D_{98\%}}{D_{p}}$$

D2 percent and D98 percents are doses received at 98 and 2% of volume coverage, respectively. The prescribed dosage is abbreviated as Dp.

The conformity index (CI) also takes into account dosage compliance in the target volume. The CI was calculated as the ratio of total volume receiving at least the prescribed dosage to target volume receiving at least the prescribed dose (Mu and Xia, 2009, Baltas et al., 1998).

(2) $$\mathrm{CI}=\frac{\mathrm{PT}V_{\mathrm{ref}}}{\mathrm{PTV}}\times \frac{\mathrm{PT}V_{\mathrm{ref}}}{V_{\mathrm{ref}}}$$

Vref describes the organ volume that lies within the prescribed isodose line. PTVref on the other hand, measures the amount of PTV which this isodose line is covers.

#### 2.2.2 Radiobiological analysis

For the 3D-CRT and JO-IMRT plans, tumor control probabilities (TCP) and normal tissue complication probabilities (NTCP) were determined.

As seen in Eq. (3) (Gay and Niemierko, 2007), NTCP is defined as follows:

(3) $$\mathrm{NTCP}=\frac{1}{1+{\left(\frac{TD_{50}}{\mathrm{EUD}}\right)}^{4\gamma_{50}}}$$

where TD_50_ stands for the point within the dose–response curve where there is a 50% probability of complication. γ_50_ is the normalized slope at the that level (Puzhakkal et al., 2016). TCP is defined as (Gay and Niemierko, 2007):

(4) $$\mathrm{TCP}=\frac{1}{1+{\left(\frac{\mathrm{TC}D_{50}}{\mathrm{EUD}}\right)}^{4\gamma_{50}}}$$

where TCD_50_ is the dose producing 50% TCP. The parameters used in the calculation are shown in Table 1.

**Table 1:** Parameters used in the TCP calculation^2^.

Structure	a	$\gamma_{50}$ (cGy)	α/β	$TD_{50}$ (cGy)	$\mathrm{TC}D_{50}$ (cGy)
PTV	−13	2,28	10	5177	7412
Brainstem	7	3	2.1	6500	7412
Spinal Cord	13	4	2	6650	7412
Parotid grand	0.5	3	2	4600	7412

#### 2.2.3 Statistical analysis

This study applied Statistical Package for the Social Sciences (SPSS) software (IBM SPSS-22, Chicago, IL). The Student's *t*-test was employed to establish statistical significance of the 3D-CRT and JO-IMRT plans. P-values <0.05 are used to determine if a difference is statistically significant.

## 3 Results

Twenty-five patients with nasopharyngeal cancer were examined utilizing a TPS (Prowess Panther,Inc, USA). When compared to 3D-CRT plans, the JO-IMRT plans showed superior tumor coverage and CI. The 3D-CRT and JO-IMRT plans had average doses to the planned target volume (PTV) of 72.1 ± 0.8 Gy and 72.5 ± 0.6 Gy, respectively (p > 0.05). Furthermore, for the 3D-CRT and JO-IMRT designs, the average doses to the parotid gland were 56.7 ± 0.7 Gy and 26.8 ± 0.3 Gy (p < 0.05), respectively. For the 3D-CRT and JO-IMRT designs, the CI and HI were 0.14 ± 0.05 and 0.13 ± 0.01 (p > 0.05), respectively; 0.73 ± 0.10 and 0.83 ± 0.05 (p < 0.05). For the JO-IMRT plan, the average TCP of PTV was 0.92 ± 0.02 and for the 3D-CRT design, it was 0.82 ± 0.08. In JO-IMRT plans, the NTCPs of the parotid glands, spinal cord, and brain stem were lower than in 3DCRT plans.

### 3.1 Dosimetric evaluation

**Fig. 1:**
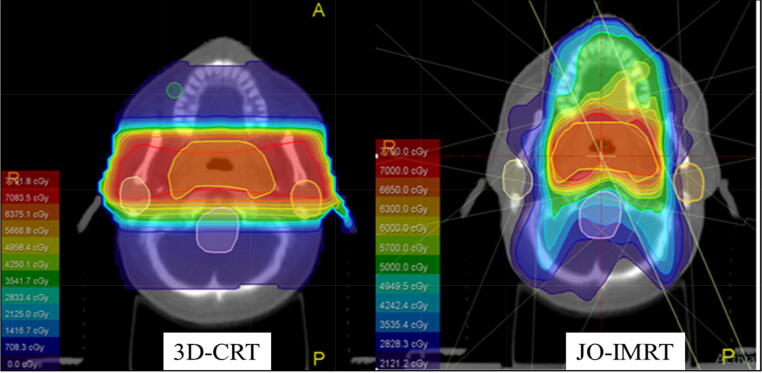
Distribution dose 3D-CRT vs JO-IMRT.

**Fig. 2:**
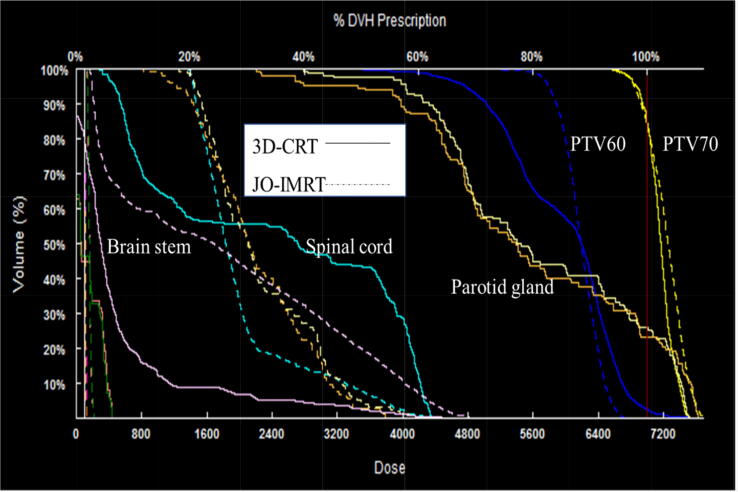
DVH comparison 3D-CRT and JO-IMRT.

**Fig. 3:**
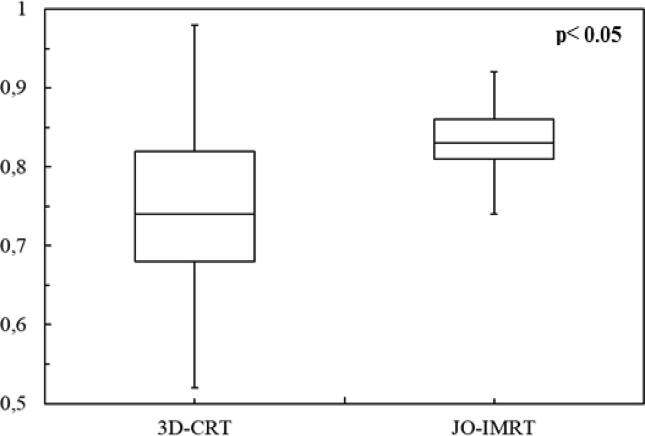
A comparison of conformity indexes (CI) between 3D-CRT and JO-IMRT.

**Fig. 4:**
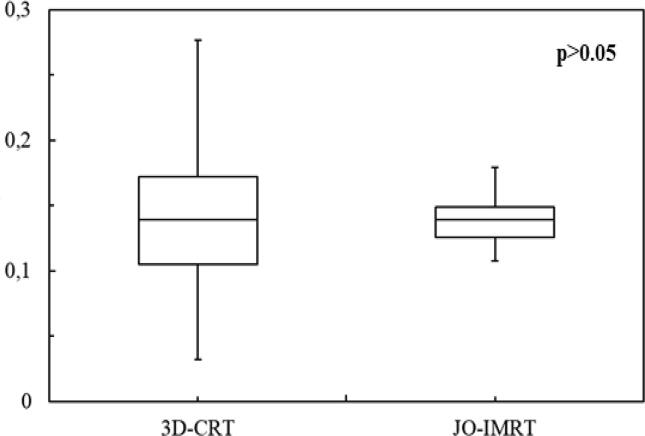
A comparison of homogeneity indexes (HI) between 3D-CRT and JO-IMRT.

The dose distribution (Fig. 1) and DVHs (Fig. 2) of the 3D-CRT and JO-IMRT plans were compared. Table 2 shows the data analysis of PTV coverage and OAR dosages for 25 patients. In terms of the highest dosage reaching the brainstem, no significant difference between the two dose distribution strategies was noted. Fig. 3, Fig. 4 provide a comparison of CI and HI between 3D-CRT and JO-IMRT.

**Table 2:** Dosimetric parameter mean values and standard deviations.

Structure	3D-CRT	JO-IMRT	p-value
	Mean ± SD	Mean ± SD	
D98 (%)	93.22 ± 4.16	94.16 ± 1.90	0.312
D95 (%)	96.64 ± 2.51	98.31 ± 1.05	0.00448
D_mean_(Gy)	72.1 ± 0.80	72.5 ± 0.60	0.649
CI	0.73 ± 0.10	0.83 ± 0.05	0.000113
HI	0.14 ± 0.06	0.14 ± 0.02	0.6047
GI	1.06 ± 0.03	1.38 ± 0.16	3.708 × 10^-10^
Spinal cord (Dmax) (Gy)	43.78 ± 1.01	41.81 ± 2.28	<0.0003
Brainstem (Dmax) (Gy)	44.62 ± 4.01	46.13 ± 2.74	0.1274
RT parotid gland (Dmean) (Gy)	56.75 ± 7.23	27.84 ± 3.29	2.2 × 10^−16^
LT parotid gland (Dmean) (Gy)	56.96 ± 6.15	27.23 ± 3.85	2.2 × 10^−16^

### 3.2 Radiobiological evaluation

**Fig. 5:**
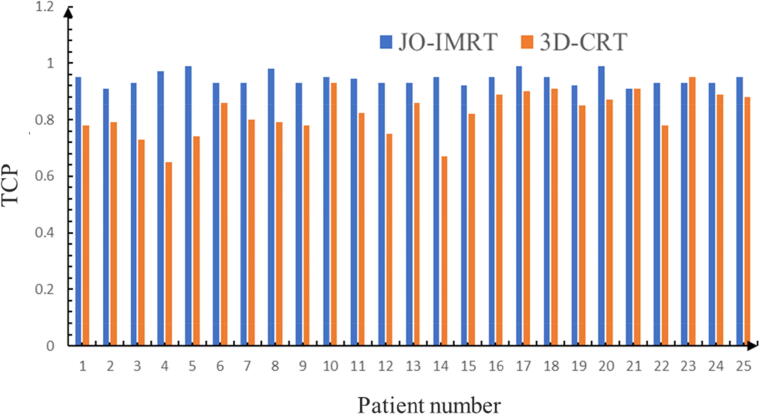
Comparison of tumor control probability between the 3D-CRT and JO-IMRT.

Fig. 5 shows the computed TCP of nasopharynx patients for the 3D-CRT and JO-IMRT plans.

## 4 Discussion

JO-IMRT was introduced about a decade ago (Tai et al., 2017b, Tai et al., 2017, Tai et al., 2018, Tai et al., 2019). This approach provides an additional IMRT option for Linacs without MLC. Since almost all modern Linacs are equipped with MLC, it is rare to see JO-IMRT implemented in clinic settings. Furthermore, JO-IMRT will usually require more treatment time than MLC-based IMRT because of the movement of jaws. This work provides extra support for using JO-IMRT in clinics, especially in some developing or undeveloped countries. This study aimed to evaluate the dosimetric and radiobiological outcomes of JO-IMRT and 3D-CRT plans.

Fig. 2, Fig. 3 reveal that the JO-IMRT plan has a superior dose distribution than the 3D-CRT plan in the target volume and can reduce dose to healthy tissues such as the spinal cord, parotid gland. This also accords with our earlier observations (Tai et al., 2017b, Tai et al., 2017, Tai et al., 2018, Tai et al., 2019).

Dose-volume histograms which relate the dose to the volume irradiated has significant explanation. For instance, on the biological effects on both the target volume PTV where the homogeneity of dose distribution may indicate better target coverage and hence better tumor control probability. Regarding the organs at risk, the effect depends on whether the organ is series or parallel, which in turn indicates the deterministic factor considered: the maximum dose received by the organs at risk (for series organs) or the volume of organs that receive a certain threshold dose (for parallel organs). Looking at Fig. 3, a larger volume receives a much higher dose by JO-IMRT. But since the brain stem is a series organ, D_max_ is the indicator. JO-IMRT delivers a higher dose to the brain stem but the difference is not high (p-value = 0.1274).

The greatest significant disparity was seen in the parotid glands, as shown in Table 2. The JO-IMRT plan improved target volume coverage, and the parotid gland dosage was much lower in the JO-IMRT plan than in the 3D-CRT plan, as shown in Table 2. For 3D-CRT and JO-IMRT, the mean dosage to the parotid gland was 56 Gy and 27 Gy, respectively. Our findings were in agreement with those of El-Ghoneimy et al. (El-Ghoneimy et al., 2012) and Puzhakkal et al. (Puzhakkal et al., 2016). Fig. 3 demonstrates that CI was better in JO-IMRT treatment plans than 3D-CRT, but there was no substantial difference in inhomogeneity index between JO-IMRT and 3D-CRT (Fig. 4). This finding is in agreement with Zheng et al. findings which showed that the planned CI of the IMRT plan (0.92 ± 0.15) was superior to the 3D-CRT plan (0.73 ± 0.12) (Zheng et al., 2015). The HI values for the 3D-CRT plans were 0.15 ± 0.05 and for the JO-IMRT was 0.13 ± 0.02. The IMRT and 3D-CRT plan have comparable HI; however, this difference is not significant statistically (p > 0.05). It concluded that the 3D-CRT technique could also produce a uniform dose distribution identical to the JO-IMRT technique.

TCP is an indication of the efficiency of the treatment in killing cancer cells. High TCP is favorable unless the harm to the nearby healthy cells is extreme. The average TCP was 94 ± 2% for the JO-IMRT and 82 ± 8% for the 3DCRT, respectively. This indicates a statistically significant difference between the two plans (p < 0.05) in favor of the JO-IMRT plans this could be attributed to the fact that the JO-IMRT plans offered much better PTV coverage and higher doses.

NTCP on the other hand indicates how much harm occurs to the normal healthy organs at risk due to treatment. The lower the NTCP, the better if appropriate target coverage is achieved. In comparison to 3D-CRT plans, the NTCP of JO-IMRT plans was much greater. The NTCP for the JO-IMRT and 3D-CRT plan were around 85% and 0%, respectively, for the appropriate parotid glands. The NTCP for the 3D-CRT and JO-IMRT designs for the left parotid glands was roughly 87% and 0%, respectively (p < 0.05). Mesbahi et al. came to the same conclusion (Mesbahi et al., 2017).

## 5 Conclusion

In comparison to the 3D-CRT approach, the JO-IMRT treatment plan was shown to increase dose coverage to the PTV, improve CI, HI, and spare the parotid glands. The outcomes of the study present a new IMRT option for Linacs without MLC. This research provides additional support for the use of JO-IMRT in clinics, particularly in developing countries. The findings show that the JO-IMRT plan has superior dose distribution in the target volume than 3D-CRT and can reduce dosage to healthy tissues such as the spinal cord and parotid gland. This is also in line with our previous observations. The majority of the OAR in the JO-IMRT plans got lower radiation doses than those in the 3D-CRT plans. In terms of radiobiology, when the JO-IMRT and 3D-CRT plans were compared, the JO-IMRT plans had a higher mean TCP than the 3D-CRT plans (p < 0.05).

## Declaration of Competing Interest

The authors declare that they have no known competing financial interests or personal relationships that could have appeared to influence the work reported in this paper.
